# Neuroimaging of acute and chronic unilateral and bilateral thalamic lesions

**DOI:** 10.1186/s13244-019-0700-3

**Published:** 2019-02-22

**Authors:** C. Tuttle, J. Boto, S. Martin, I. Barnaure, A. M. Korchi, M. Scheffler, M. I. Vargas

**Affiliations:** 1 Division of Radiology, Faculty of Medicine, Geneva University Hospital, Geneva, Switzerland; 2 Division of Neuroradiology, DISIM, Faculty of Medicine, Geneva University Hospital, Rue Gabrielle-Perret-Gentil 4, 1211 Geneva 14, Switzerland

**Keywords:** Thalami, Magnetic resonance imaging, Computed tomography, Deep brain stimulation, Infection, Neoplasia, Vascular and ischaemic diseases

## Abstract

The thalami are bilateral ovoid grey matter cerebral structures bordering the third ventricle on both sides, which participate in functions such as relaying of sensory and motor signals, regulation of consciousness, and alertness. Pathologies affecting the thalami can be of neoplastic, infectious, vascular, toxic, metabolic, or congenital origin.

The purpose of this review is to provide a comprehensive approach to the thalamus focusing on its anatomy, the main pathologies affecting this structure and their radiological semiology on CT and MRI. We will also illustrate the importance of multimodal MR imaging (morphologic sequences, diffusion-weighted imaging, perfusion, spectroscopy) for the diagnosis and treatment of these conditions.

## Key points

- Multimodal MRI plays a crucial role in the detection and characterisation of thalamic lesions
- Bilateral thalamic lesions should point to venous thrombosis, which is reversible if treated early
- MR imaging is crucial for guiding stereotactic surgical positioning of DBS electrodes
- The main differential diagnosis of bilateral thalamic lesions are tumour, ischaemia, and venous thrombosis

## Introduction

The thalamus is a complex structure involved in several cognitive, motor, and sensory processes. Interactions between the thalamus and cortical areas are well known. Thalamic pathologies can be classified into neoplastic, metabolic, congenital, vascular, and infectious. Multimodal MR imaging, which includes morphologic sequences, diffusion-weighted imaging, and vascular and perfusion imaging as well as spectroscopy, is useful for the diagnosis and management of these conditions. CT also plays an important role and often complements MRI.

## Anatomy and physiology of the thalamus

The two thalami are paired ovoid deep brain structures composed mainly of grey matter, located in the dorsal diencephalon together with the epithalami and the metathalami. In some classifications, the metathalami, also known as the geniculate bodies, are considered part of the thalami.

The thalami process and relay information through afferent and efferent fibers. They play a key role in the integration of sensory information (except for the sense of smell), as well as in motor and cognitive functions (behaviour, memory, emotions, sleep-wake cycle, executive functions, and alertness).

Each thalamus is approximately 3.5 cm long, and both thalami are linked across the midline by a thin interthalamic adhesion, also composed of grey matter [1]. The thalami form most of the wall of the third ventricle (their anterior tubercles outline the posterior wall of the foramina of Monro), and cranially, they shape part of the floor of the lateral ventricles. Laterally, they are delineated by the internal capsules, and inferiorly, they are separated from the mesencephalon by the subthalami. The posterior part of the thalami is known as the pulvinar, which is in close proximity to the superior and inferior colliculi in the tectal plate with the geniculate bodies situated in-between. Cranially, the fornices form a groove on the pulvinars. Patients with lesions affecting the posterior thalamus may present with variable sensory deficits, weakness, memory impairment, aphasia, hand tremor, and dystonia. More specifically, if the inferolateral part of the pulvinar is affected, hemianopsia and quadrantanopsia can be found [2].

The thalami include several nuclei. The anterior nucleus is the most anteriorly located. Conditions associated with this nucleus result in diencephalic amnesia, from which the degree of recovery is variable [3]. The medial part of the thalamus is composed of a large medial dorsal and a small medial ventral nucleus. Laterally, the thalamus is subdivided into a dorsal and a ventral tier of nuclei. The dorsal tier includes the lateral dorsal and lateral posterior nuclei, as well as the pulvinar. The ventral tier of the lateral part comprises the ventral anterior, ventral lateral, and ventral posterior nuclei (ventral posteromedial (VPM) and ventral posterolateral (VPL) nuclei). There are three different clinical syndromes associated with lesions affecting the lateral thalamus. The first one is characterised by hemisensory loss, hemiataxia, and involuntary movements. The second and third ones include pure sensory and sensory-motor deficits, respectively [4]. In addition to these, there are other thalamic nuclei, including the intralaminar, midline, and reticular nuclei. The metathalamus, composed of the medial and lateral geniculate bodies, is sometimes considered part of the thalamus.

From a functional point of view, only the ventral tier nuclei of the lateral part of the thalamus and the geniculate bodies are considered to be specific. A lesion affecting the geniculate bodies will cause homonymous hemianopsia or quandrantanopsia [5]. The VPM receives sensory information from the head and face through the trigeminal lemniscus and tastes information through the solitary tract. The VPL receives exteroceptive sensory information (pain, touch, temperature) from the contralateral side of the body by the ascending spinothalamic tract (spinal lemniscus) and proprioceptive information (sense of the relative position of body parts and of muscle strength) through the medial lemniscus (cuneate and gracile nuclei). Motor information is also received from the cerebellum (via the superior cerebellar peduncle to the ventral lateral nucleus) and the corpora striata. The geniculate bodies form part of the hearing and vision pathways. These specific nuclei act as gateway and relay stations before the information is passed onto the ipsilateral sensory cortex, in particular by the thalamocortical tracts (to sensory Brodmann areas 1, 2, and 3), passing through the posterior limb of the internal capsule.

**Fig. 1:**
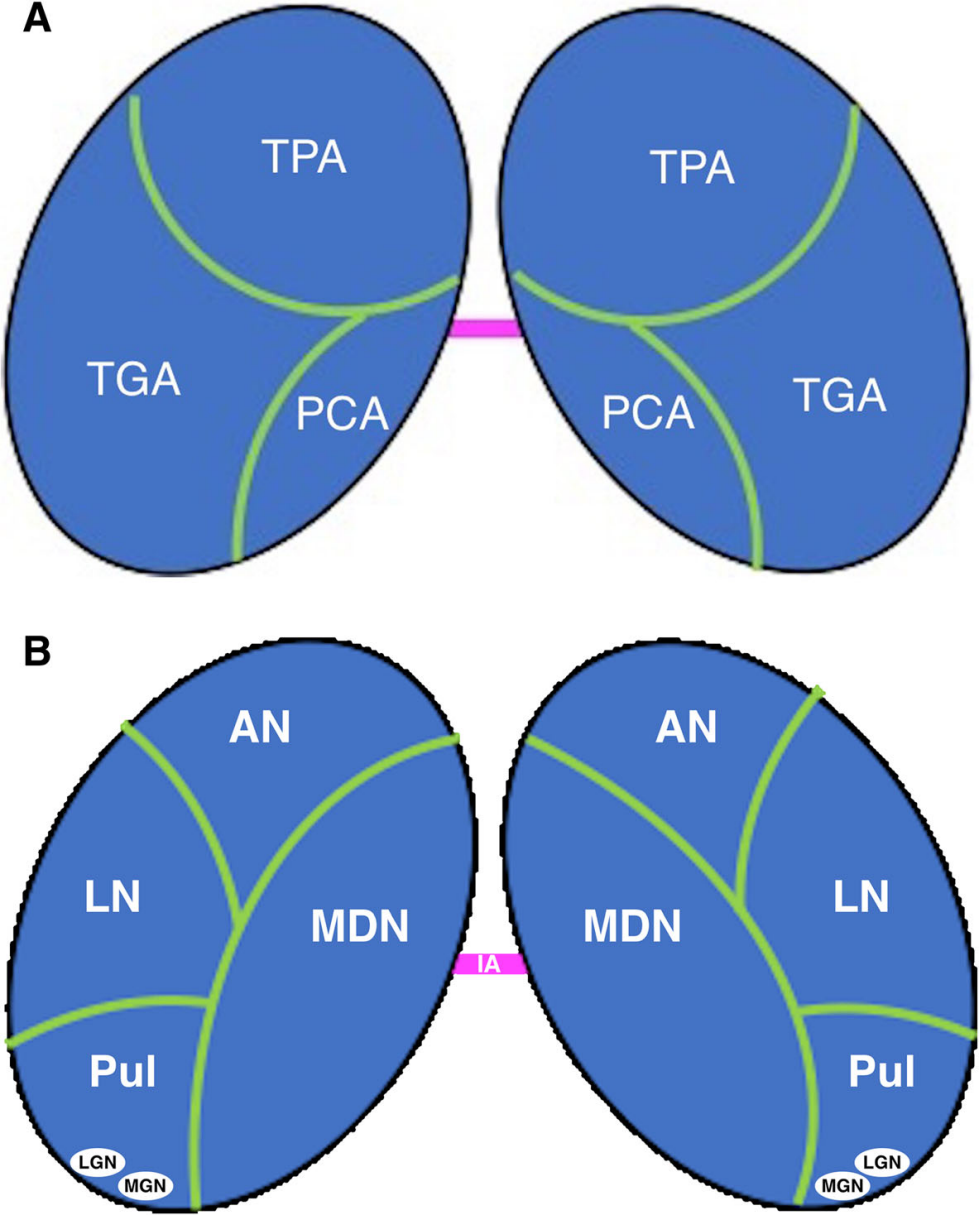
Diagrams show the different anatomic regions of the thalamus (**a**) and its vascularisation (**b**). TPA, thalamoperforating arteries; TGA, thalamogeniculate arteries; PCA, posterior choroidal artery; AN, anterior nucleus; LN, lateral nuclei; MDN, mediodorsal nuclei; Pul, pulvinar; LNG, lateral geniculate nucleus; MGN, medial geniculate nucleus

Non-specific nuclei include the anterior nucleus, implicated in cognitive functions, such as attention and short-term memory, and the medial dorsal nucleus, which is associated with mood. Here, connections exist with the limbic system. The relative position of the thalamic nuclei is illustrated in Fig. 1a.

Vascularisation is assured by branches of the posterior cerebral artery (thalamoperforating branches for the anterior thalamus and thalamo-geniculate branches for the posterior portions), the posterior communicating artery (anteroinferior territory), and the posterior choroidal arteries stemming from the P2 segment of the posterior cerebral artery. The artery of Percheron, originating from one of the two P1 segments, is a normal variant and supplies the posteromedial aspect of both thalami (Fig. 1b). Venous drainage is through the deep venous system into the internal cerebral veins and the basal veins of Rosenthal.

## Pathology

### Primary tumours

#### Glioma

**Fig. 2:**
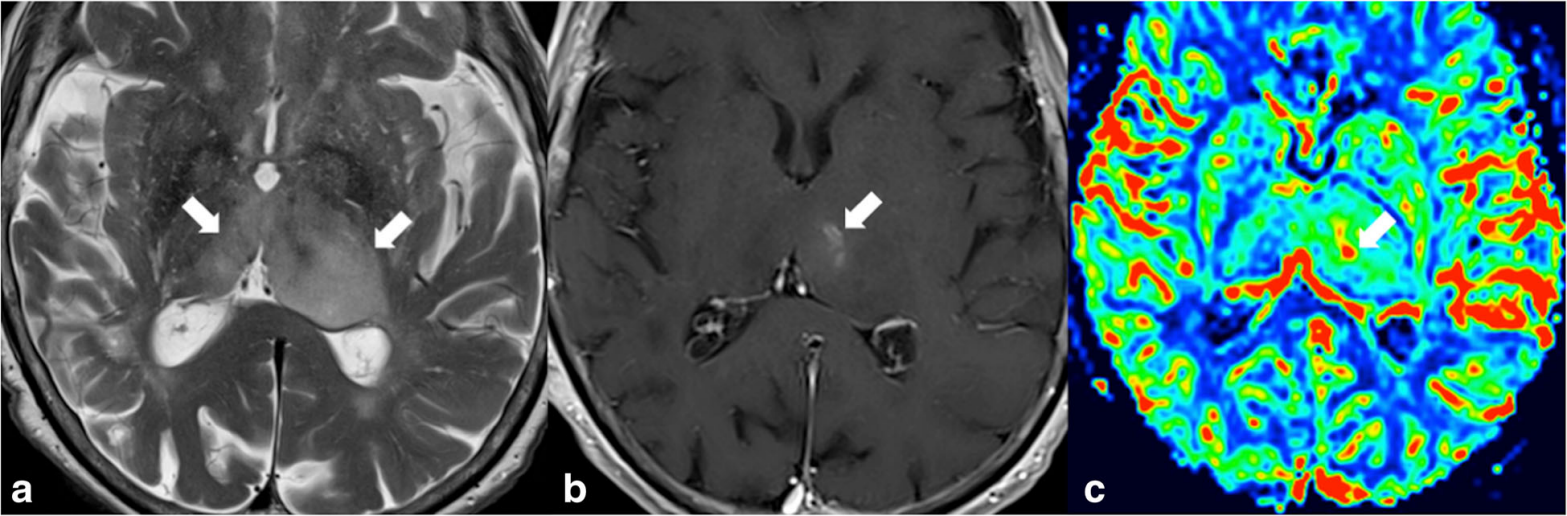
T2WI hyperintense lesions in the thalami (arrows in **a**), with mass effect on the third and left lateral ventricles, showing faint enhancement on the left thalamus (arrow in **b**). Note that the hypervascularisation on the CBV map sequence (arrow in **c**), reflecting neoangiogenesis, perfectly matches the enhancing component of the lesion, which corresponded to a biopsy-proven high-grade glioma

Primary tumours of the thalamus (Fig. 2) represent 1% of all primary brain tumours [6] of which the majority are gliomas. On MRI, these lesions are hyperintense on T2WI and hypointense on T1WI. Low-grade gliomas show no enhancement [7].

Enhancement, hyperperfusion, central necrosis, and haemorrhage are seen in high-grade lesions (grades III and IV). Diffusion restriction is seen in hypercellular tumours. Spectroscopy often shows increased choline, choline/creatine ratio and myoinositol, and decreased NAA. In high-grade glioma, there may be a lactate and lipids peak reflecting necrosis [8].

#### Lymphoma

**Fig. 3:**
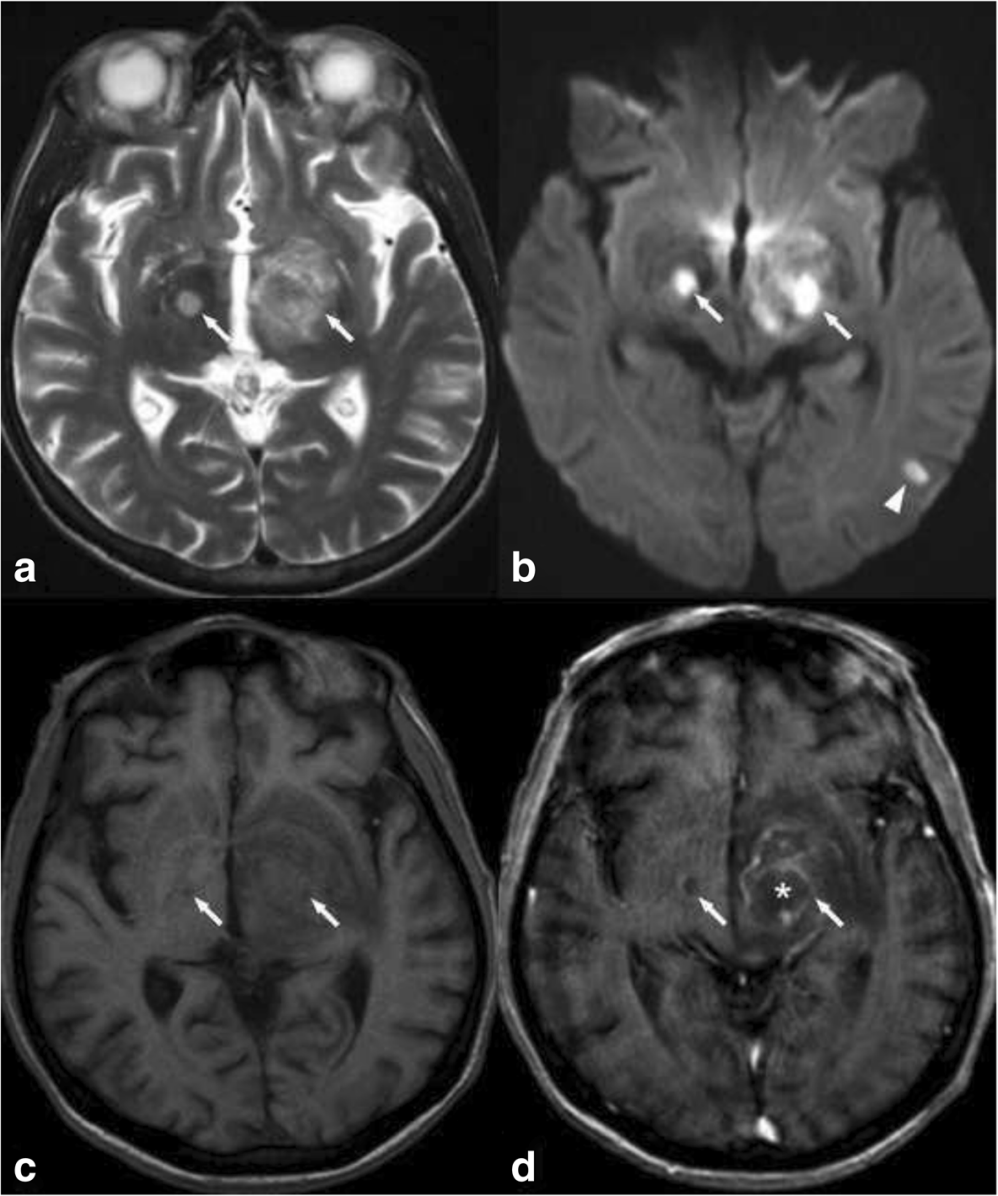
T2WI and DWI hyperintense lesions in both thalami (arrows in **a** and **b**). The lesions are hypointense on T1, and there is a non-enhancing central region corresponding to an area of necrosis (* in **d**). This pattern of enhancement suggests lymphoma in immunocompromised patients

Primary CNS lymphoma is a rare form of non-Hodgkin lymphoma [9], and it affects both immunocompetent and immunocompromised patients. The most commonly affected regions in CNS lymphoma are the cerebral hemispheres. The corpus callosum, periventricular white matter, the basal ganglia, and the thalami are also frequently affected. CT shows high-attenuation lesions with strong enhancement. On MRI, lesions are hypointense to isointense to grey matter on T2W and T1W imaging with restricted diffusion due to high cellularity. Homogeneous enhancement is seen in immunocompetent patients, whereas ring enhancement and central necrosis are found in immunocompromised patients (Fig. 3). MR spectroscopy usually shows elevated choline levels. Perfusion imaging produces a characteristic curve with overshooting of the baseline in the recovery phase [10].

### Metastases

**Fig. 4:**
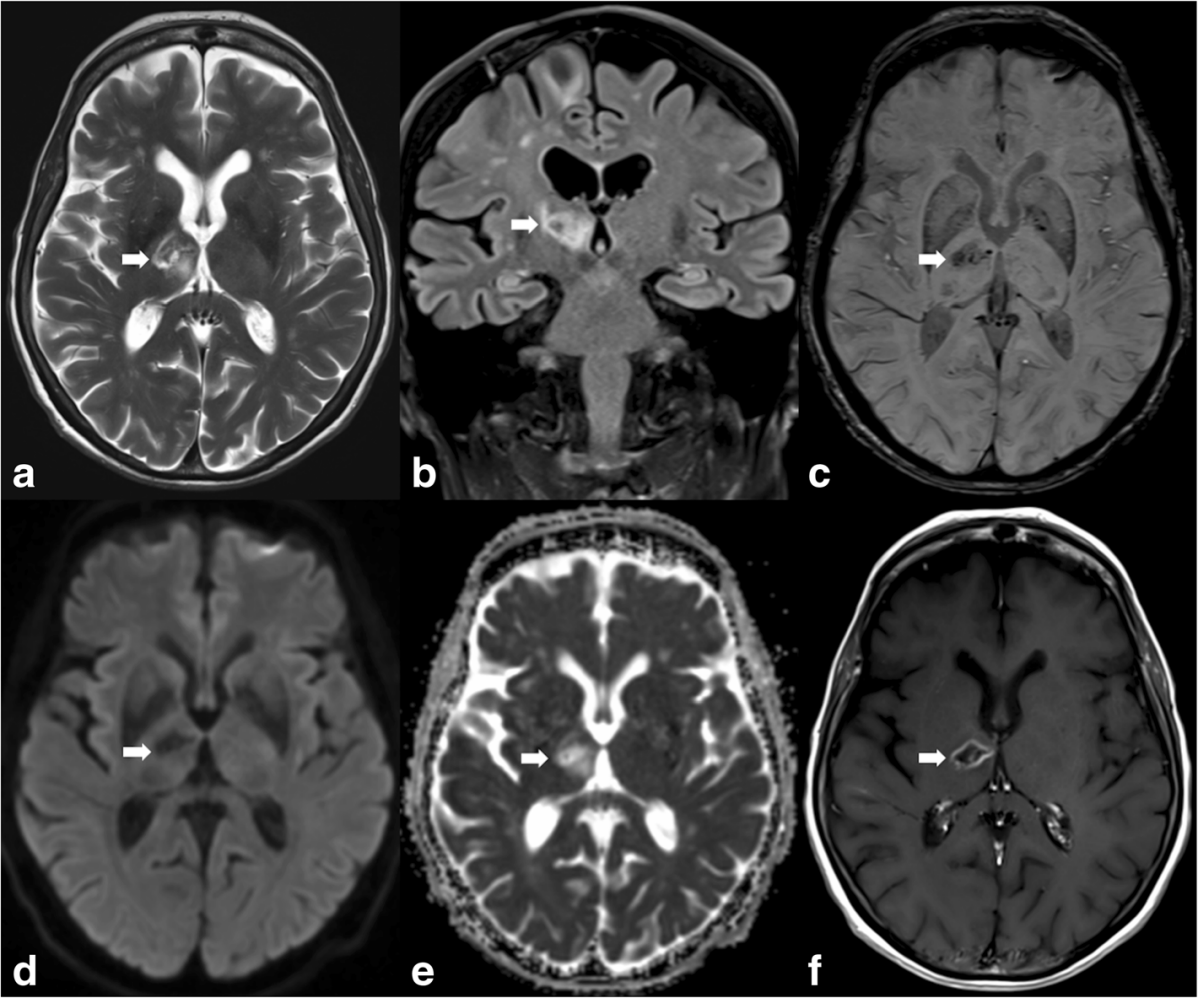
MRI illustrates a T2WI and FLAIR hyperintense lesion in the right thalamus and posterior limb of the internal capsule (arrows in **a** and **b**). There is a small haemorrhagic component seen on SWI (arrow in **c**). No diffusion restriction is observed (arrows in **d** and **e**), and there is ring enhancement (arrow in **f**)

Metastatic disease should be suspected in patients with a known primary tumour and when multiple lesions are seen. Lesions can be haemorrhagic and/or necrotic and show variable patterns of enhancement (Fig. 4).

### Metabolic disease

#### Wernicke’s encephalopathy

**Fig. 5:**
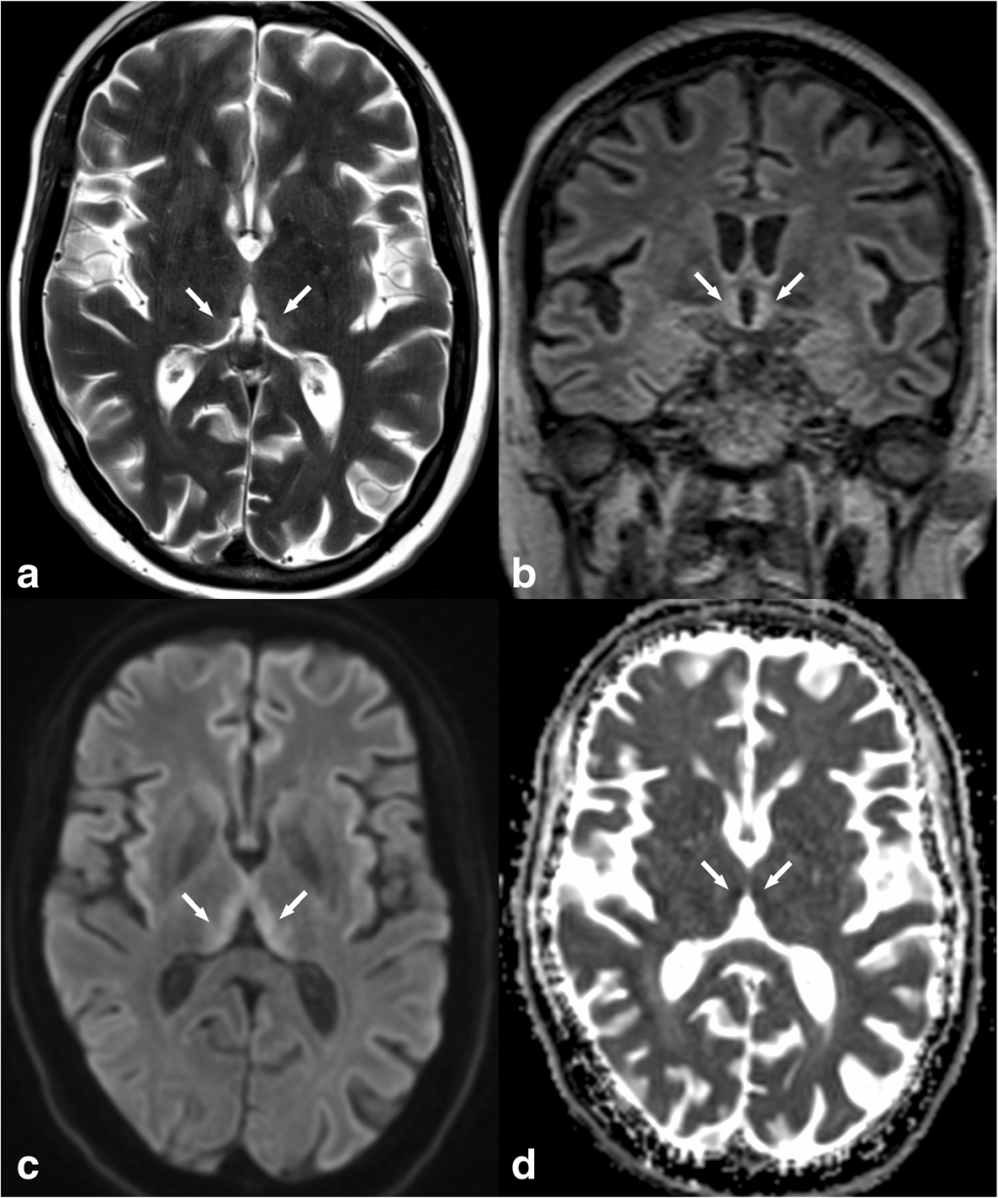
Axial FSE T2 (**a**) and coronal FLAIR (**b**) illustrate hyperintensity surrounding the third ventricle (arrows). These abnormalities are associated with diffusion restriction (arrows in **c** and **d**)

Wernicke’s encephalopathy, or alcoholic encephalopathy, results from dietary deficiency of vitamin B_1_. The classic triad of symptoms includes altered consciousness/confusion, ocular dysfunction, and gait disturbances/ataxia. Approximately 50% of Wernicke’s encephalopathy cases are not alcohol related and are caused by malnutrition and intractable vomiting [11], among others. MRI shows T2WI and FLAIR hyperintensity, T1 hypointensity, and gadolinium enhancement adjacent to the third ventricle (Fig. 5), the tectal plate and the periaqueductal area, hypothalamus, medial thalami, mammillary bodies, and the nucleus of cranial nerve XII [12]. These regions are at increased risk in alcoholic patients possibly due to malfunction of the blood-brain barrier.

#### Fahr’s disease

**Fig. 6:**
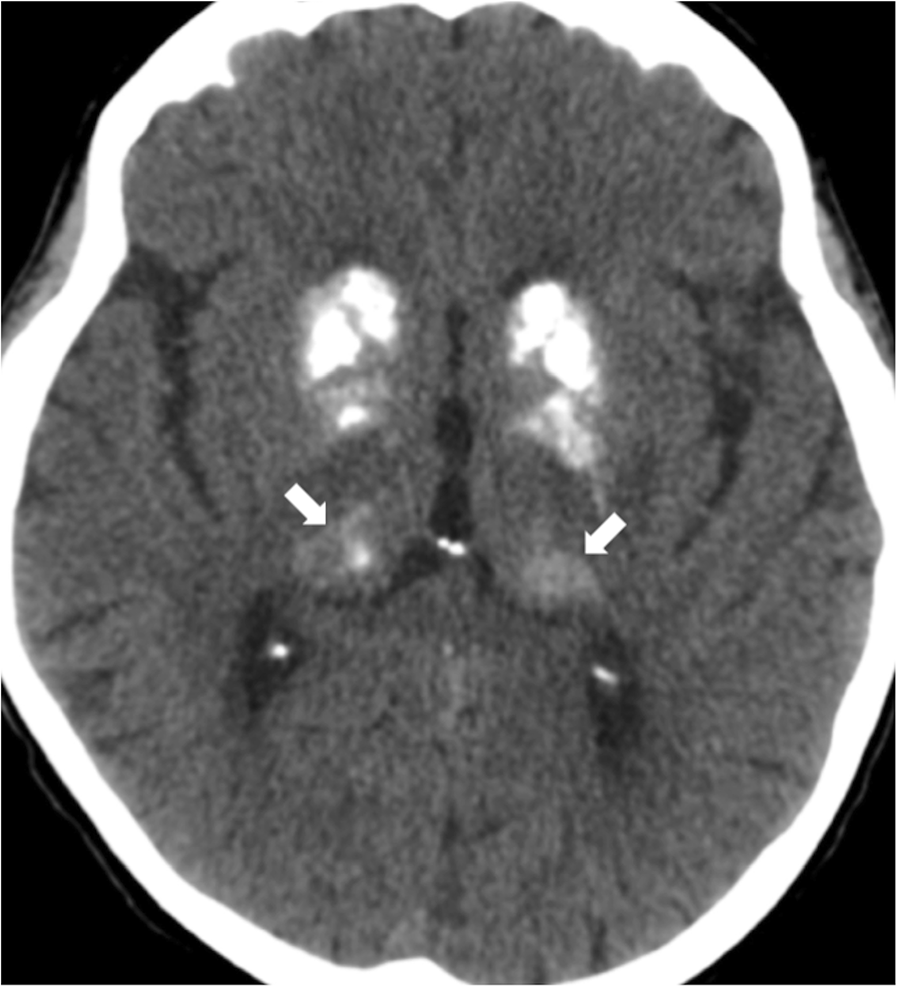
High attenuation in the basal ganglia and thalami (arrows) on non-contrast CT due to calcifications in a patient with Fahr’s disease

Fahr’s disease is a degenerative condition involving the basal ganglia bilaterally and symmetrically, including the thalamus, dentate nuclei, and deep or subcortical white matter (Fig. 6). It shows, in most cases, autosomal recessive or dominant transmission. Clinical manifestations include pseudoparkinsonism, dystonia, and neuropsychiatric disturbances. The main differential diagnosis includes Aicardi-Goutières encephalopathy, Cockayne syndrome, hyperparathyroidism, and complications of intrathecal chemotherapy and radiotherapy.

CT shows calcification of the basal ganglia, thalami, and subcortical white matter. MRI shows variable signal intensity on T1WI and T2WI in the same locations.

#### Wilson’s disease

**Fig. 7:**
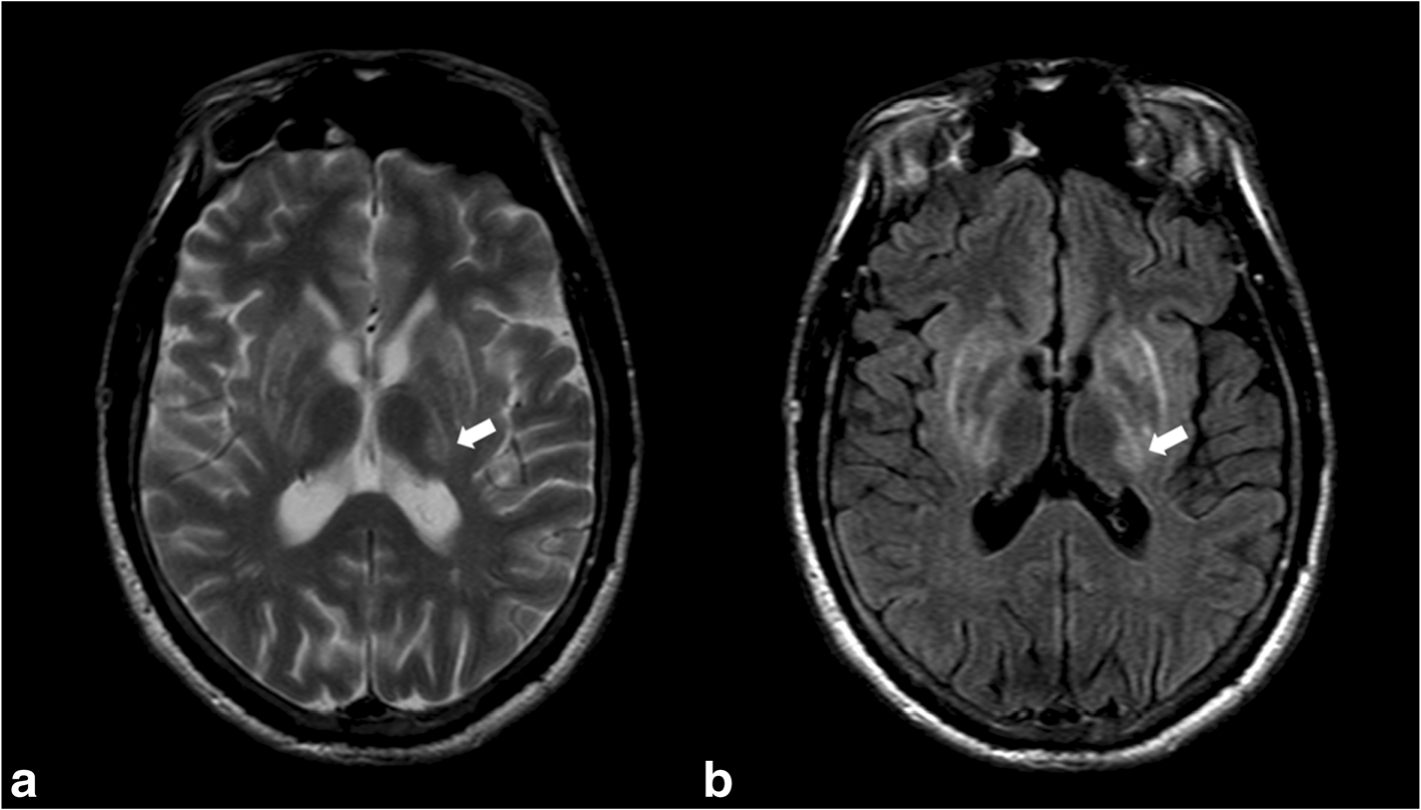
High signal intensity of the basal ganglia and left thalamus (arrows) on FSE T2WI (**a**) and FLAIR (**b**) in a patient known for Wilson’s disease

This autosomal recessive condition leads to deposits of copper in the liver and brain. Clinical symptoms include dysarthria, dystonia, and tremor. On MRI, copper deposits in the lentiform nucleus and thalamus are hypointense on T2WI and gradient echo (GRE). High signal intensity in the outer rim of the putamen is often seen on T2WI and FLAIR (Fig. 7).

### Congenital disease

#### Neurofibromatosis type 1

Neurofibromatosis type 1 (NF1) is an autosomal dominant disease characterised by multiple neurofibromas. Focal areas of signal intensity (FASIs) of the white matter, basal ganglia, and thalamus are found in this pathology. Possible causes include transitional myelinopathy with vacuolation of the myelin sheath, dysplasia, and hamartomatous lesions.

**Fig. 8:**
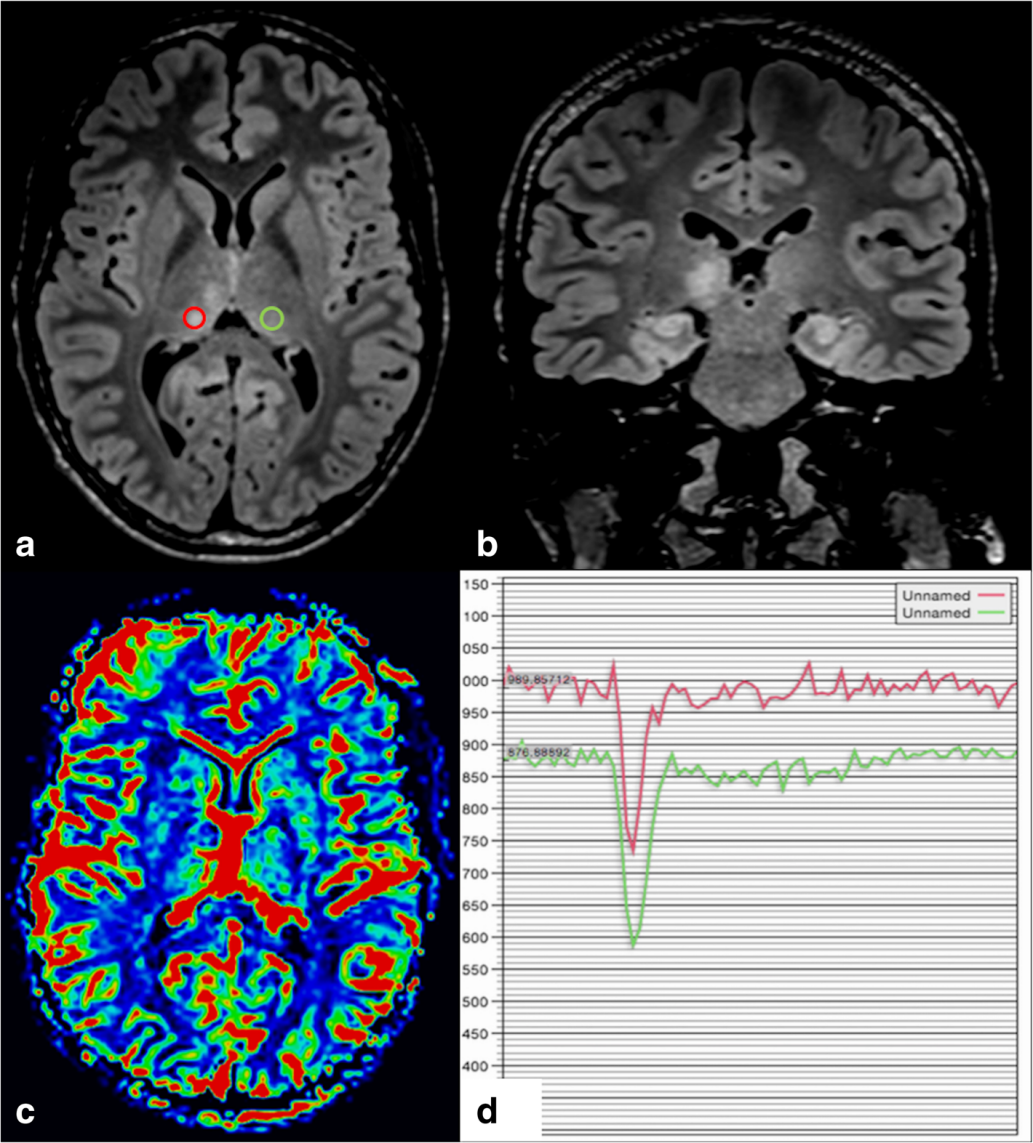
Bright confluent foci in the right thalamus on axial and coronal (**a**, **b**) FLAIR, not associated with mass effect or surrounding oedema. Absence of hypervascularisation on perfusion MR (**b**, **c**) further confirms the benign nature of these lesions allowing differentiation between primary brain tumour such as astrocytoma or glioblastoma and benign lesions. These are FASIs of a patient with NF1

As in other locations, lesions in the thalamus appear as bright foci on T2WI and FLAIR, isointense to hyperintense on T1WI, and typically show no contrast enhancement. They are not associated with mass effect or peripheral oedema. Spectroscopy and MR perfusion may be useful for distinguishing NF1 lesions from glial tumour, which is the main differential diagnosis (Fig. 8). Although FASIs and tumours both show increased Cho:Cr ratio, which is higher in tumours, FASIs have near-normal NAA (*N*-acetylcholine) whereas tumours have markedly decreased NAA. Concerning perfusion, tumours are hyperperfused whereas FASIs will show similar or decreased perfusion relative to normal brain.

### Vascular

#### Haemorrhage

**Fig. 9:**
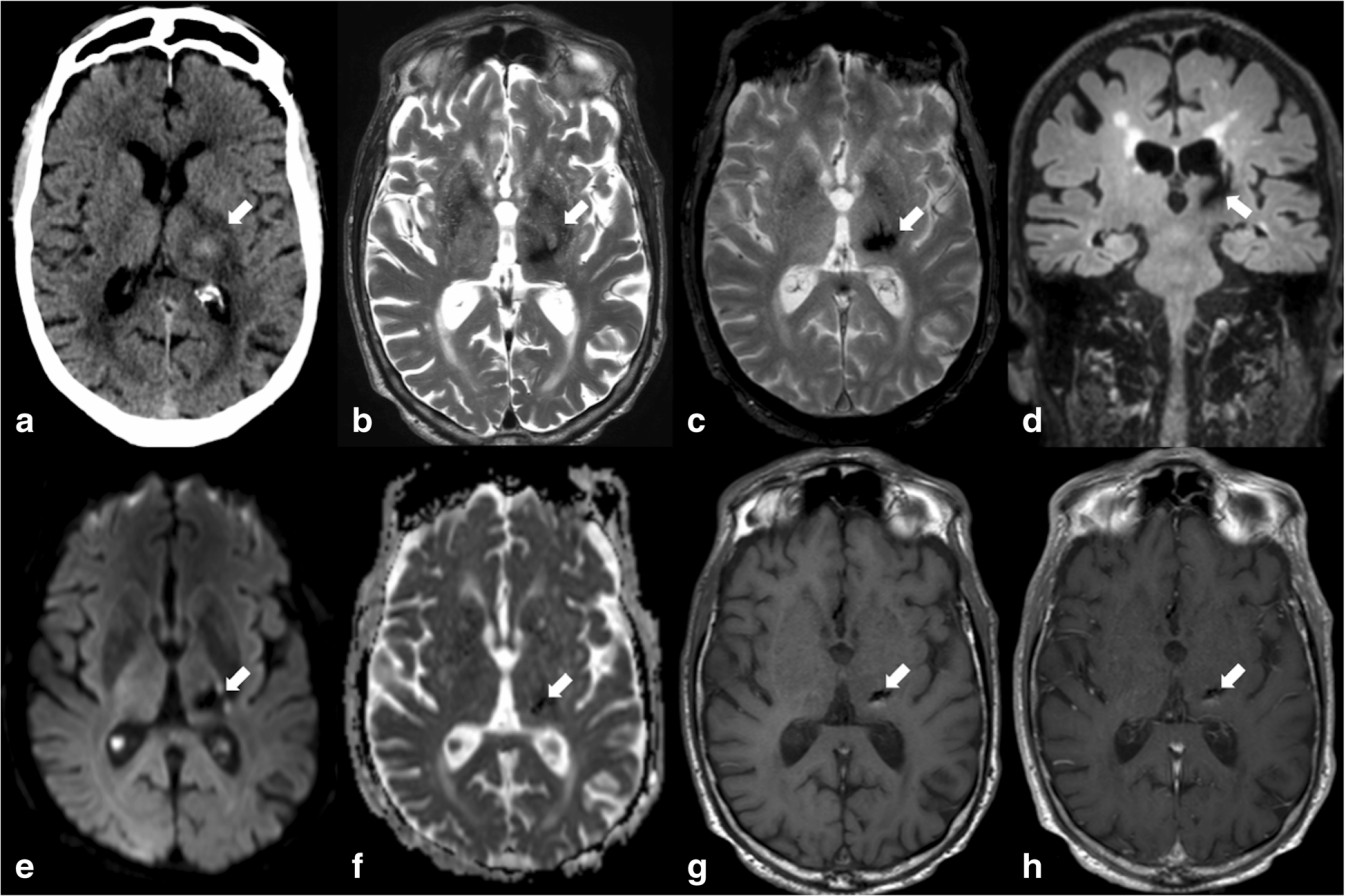
There is a hyperdense lesion on non-contrast surrounded by oedema (arrow in **a**). Follow-up MRI shows resolution of the oedema and low signal intensity of the lesion on all sequences including T2 (**b**), T2* (**c**), FLAIR (**d**), DWI b1000 (**e**), ADC map (**f**), non-contrast T1 (**g**), and T1 post contrast (**h**) reflecting the expected progression of a subacute into chronic haematoma in a patient with hypertension

Causes of brain haemorrhage include hypertension, cerebral amyloid angiopathy, tumour (primary and secondary), and vascular malformations. Intraparenchymal haemorrhage shows high attenuation on CT in the acute and subacute stages. The attenuation progressively decreases until it becomes lower than normal brain in the chronic stages. On MRI, haemorrhage shows variable signal intensity depending on the age of the blood products. Susceptibility-weighted imaging (SWI) is a magnetic susceptibility sequence which includes both magnitude and phase imaging and is therefore able to distinguish haemorrhage from calcifications. Hypertension is the most common cause of intracerebral haemorrhage, and typical locations are the basal ganglia, thalamus (Fig. 9), pons, and cerebellum.

#### Arteriovenous malformation

Arteriovenous malformations (AVMs) are considered congenital lesions, and intracerebral haemorrhage is the most common and serious presentation [13]. Symptoms includes headache, neurological deficits (motor or visual), and epilepsy. AVMs in the deep basal ganglia and thalami are at higher risk of bleeding because of the contribution of small perforating arteries to the vascularisation of these structures and also due to the deep venous drainage.

**Fig. 10:**
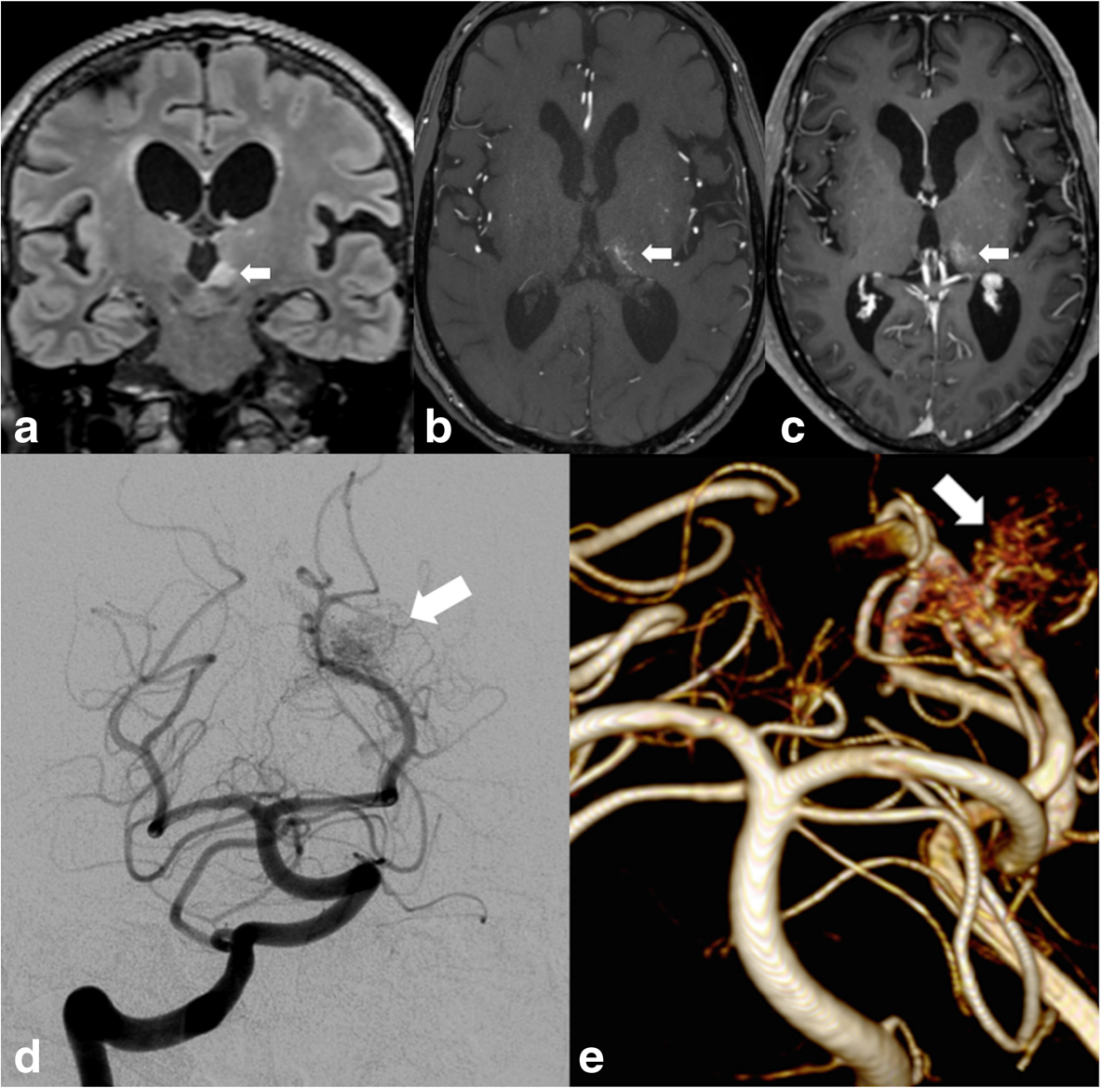
High signal intensity on FLAIR in the left thalamus (arrow in **a**) associated with a “bag of worms” appearance on 3D TOF (arrow in **b**). DSA and 3D TOF reformat (**e**) demonstrate abnormal vessels corresponding to the nidus of an AVM (arrows in **c** and **d**)

AVMs appear as heterogeneous lesions on T2WI and T1WI with flow voids best appreciated on T2WI and usually likened to a “bag of worms”. Dynamic MRA plays an essential role in demonstrating the arterial supply, the nidus, and the venous drainage of these lesions (Fig. 10).

#### Arterial ischaemic lesion

**Fig. 11:**
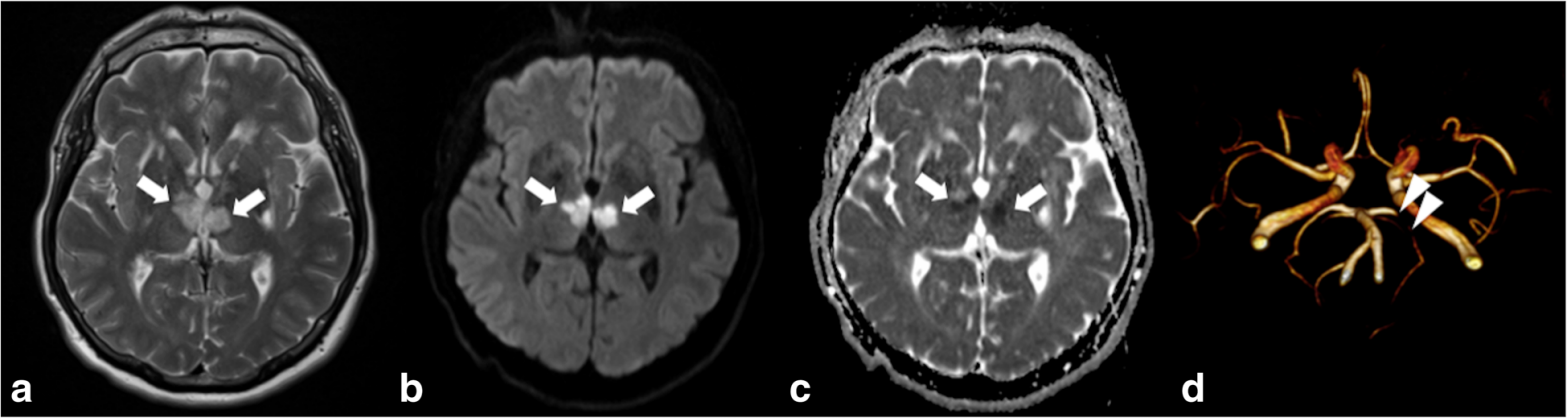
High signal intensity of both thalami (arrows in **a**) associated with diffusion restriction in the same area (arrows in **b** and **c**) consistent with ischaemia due to occlusion of the artery of Percheron (anatomic variant). 3D TOF shows stenosis of the P1 segment of the left posterior cerebral artery (arrowheads in **d**). The Percheron artery itself is not visible

Thrombosis of arteries supplying the thalamus is responsible for unilateral or bilateral ischaemic events. In the acute phase, ischaemia appears as low attenuation on CT due to cytotoxic oedema and reduced cerebral blood flow. A high-attenuation thrombus in the obstructed artery may be seen on non-contrast CT (dense artery sign) and as low signal intensity on T2* or SWI sequences. CTA will show an arterial filling defect at the site of thrombosis. MRI shows unilateral or bilateral lesions (in the case of thrombus in the artery of Percheron) (Fig. 11) seen as diffusion restriction and hyperintensity on T2WI and FLAIR [14, 15]. The main differential diagnosis includes venous infarct and tumour.

#### Hypoxia/ischaemia

**Fig. 12:**
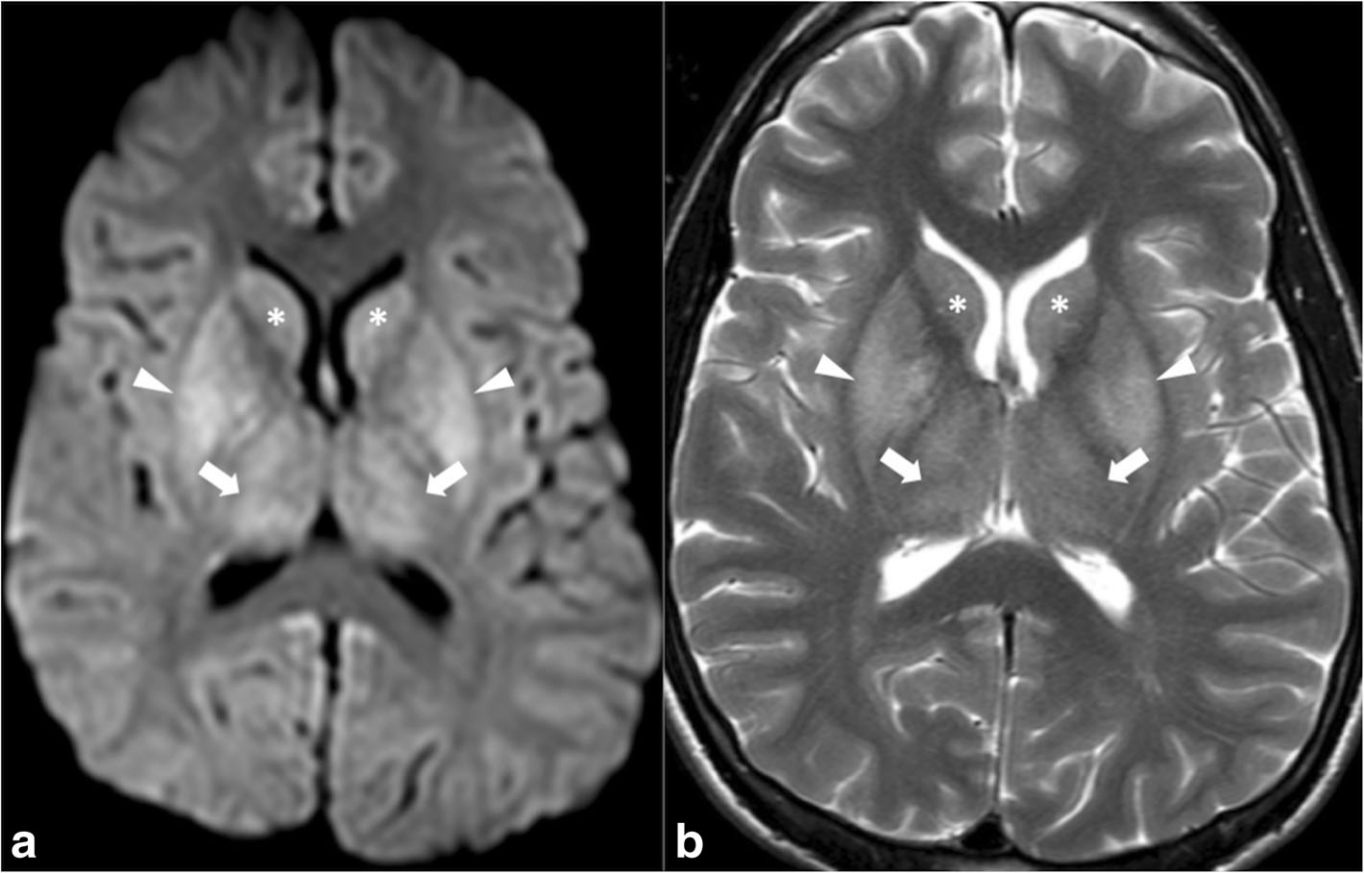
DWI illustrates hyperintensity of both thalami (arrows in **a**), the lentiform nuclei (arrowheads in **a**), and caudate (asterisk in **a**) following cardiac arrest. Note hyperintensity in the same regions on T2WI (**b**) corresponding to ischaemia due to hypoxia

In low-output states, ischaemic lesions initially occur at specific locations usually referred to as the watershed areas, which are located between two adjacent arterial territories. Structures in these locations, which include the thalami, basal ganglia, and deep white matter, receive part of their blood supply through end arteries (Fig. 12). The relative paucity of collaterals in these regions explains why they are particularly sensitive to ischaemic events.

CT and MRI findings are the same as for ischaemia caused by arterial occlusion, but the distribution of the lesions does not follow an arterial territory. In severe asphyxia, the “reversal sign” may be seen on CT, in which white matter shows higher attenuation than grey matter.

#### Venous thrombosis

Thrombosis of veins draining the thalamus (internal cerebral veins and basal veins of Rosenthal) leads to venous congestion with the development of infarction, oedema, and/or haemorrhage, usually in both thalami. The differential diagnosis includes ischaemia, due to occlusion of the artery of Percheron, and tumour. Venous thrombosis causes sudden onset of symptoms and is rapidly progressive. It should be suspected in patients with risk factors, which include head trauma, pregnancy, oral contraception, and hypercoagulable states.

**Fig. 13:**
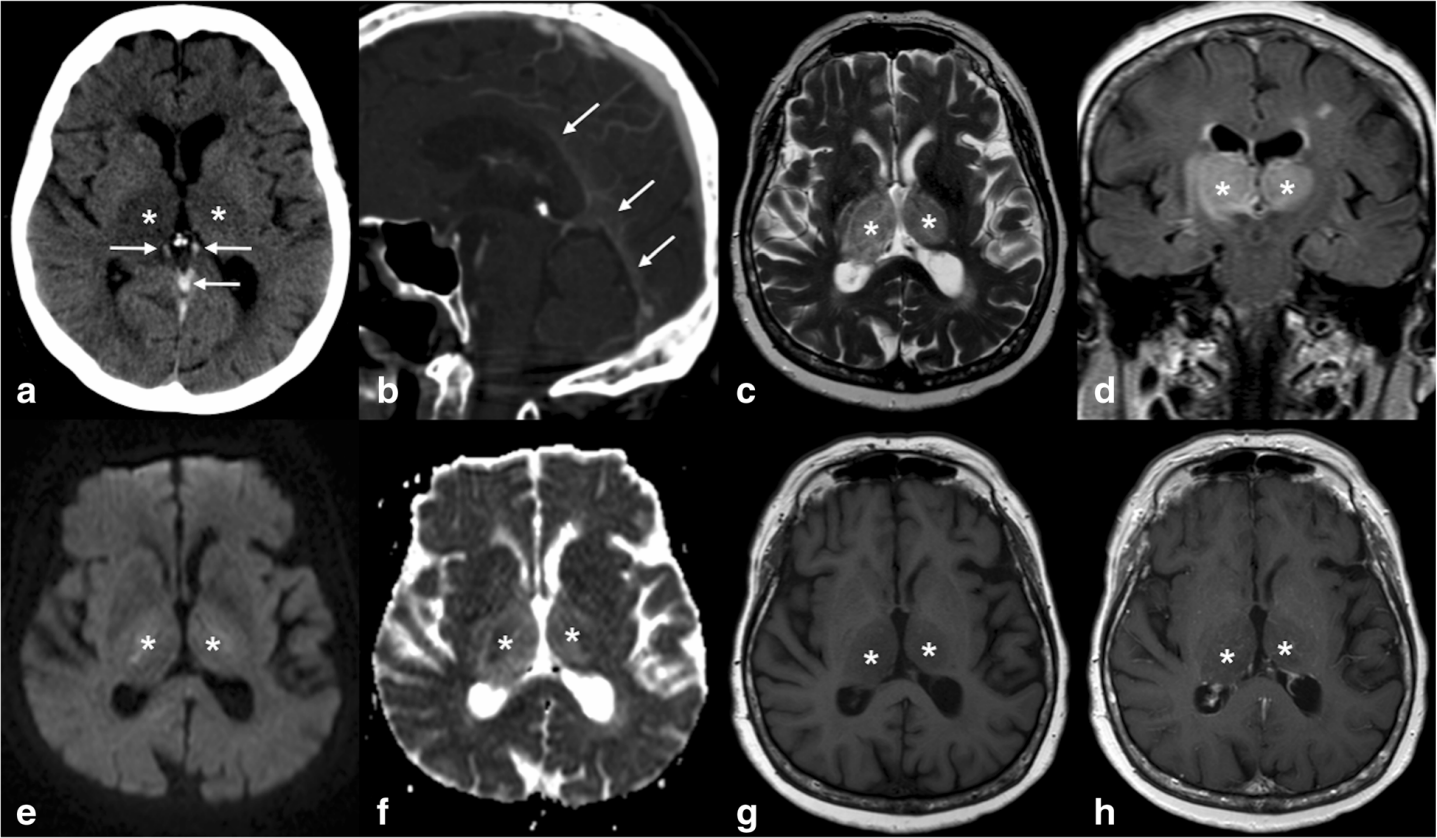
Non-contrast CT shows high attenuation of the internal cerebral veins and the vein of Gallen (arrows in **a**). CTV illustrates filling defect in the inferior sagittal sinus, vein of Gallen, and straight sinus (arrows in **b**). Both thalami (asterisk) are hypodense on CT (**a**), hyperintense on T2 (**c**) and FLAIR (**d**), hypointense on T1 (**g**), and show no enhancement (**h**). There is no diffusion restriction (**e**, **f**) reflecting vasogenic oedema secondary to venous thrombosis

Non-contrast CT shows low attenuation in the thalamus and/or linear high attenuation of the internal cerebral veins. In the early stages of venous thrombosis, MRI shows T2WI, FLAIR, and DWI hyperintensity with high ADC values reflecting vasogenic oedema from venous congestion. This often progresses to acute ischaemia which will cause diffusion restriction. Contrast-enhanced CTV and MRV are helpful to depict the thrombus and confirm the diagnosis of venous thrombosis (Fig. 13).

### Infection

**Fig. 14:**
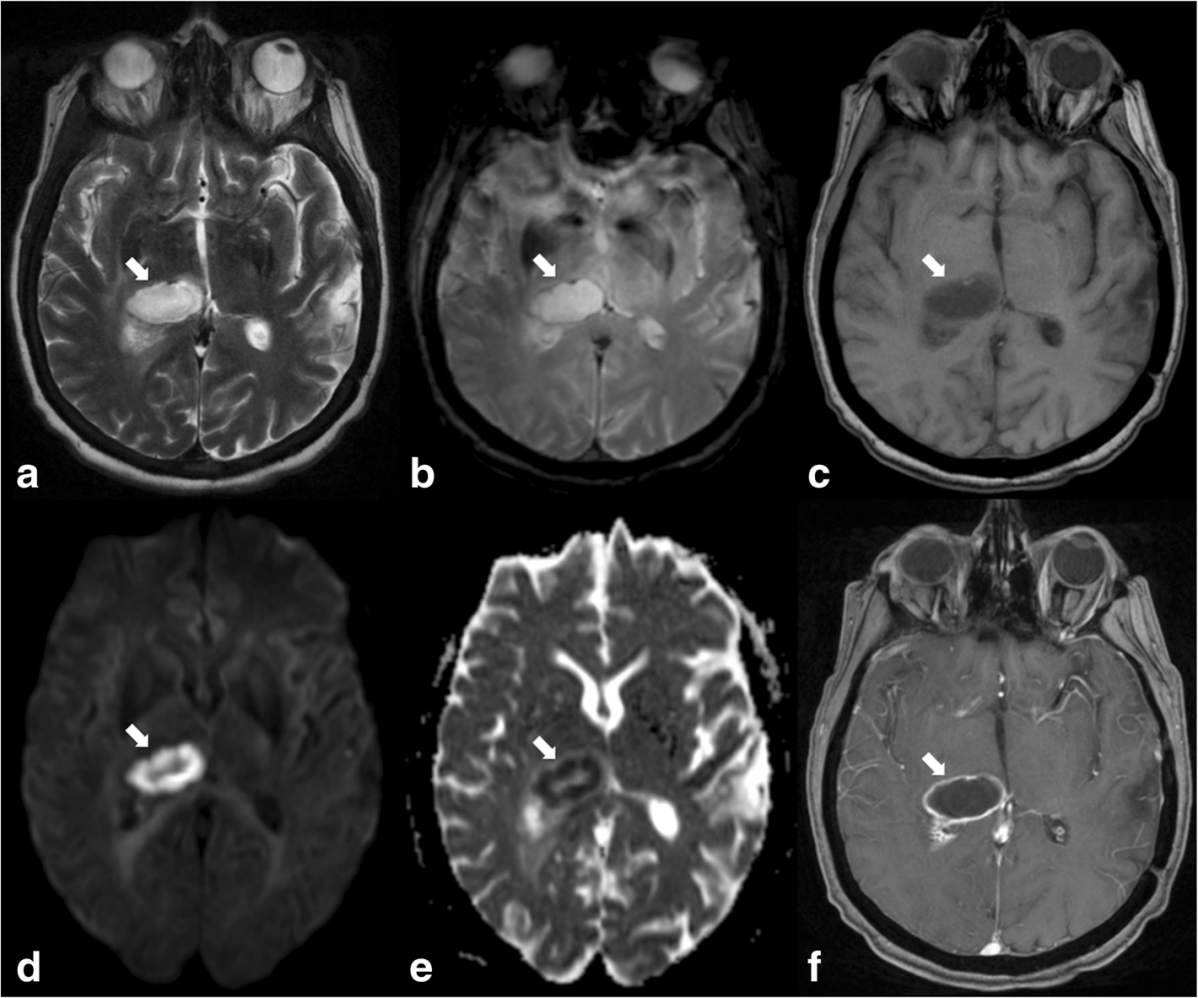
Ovoid lesion in the right thalamus producing mass effect on the third ventricle, which is hyperintense on T2 (arrow in **a**), shows slight peripheral low signal intensity on T2* (arrow in **b**), is hypointense on T1 (arrow in **c**), and shows diffusion restriction (arrows in **d** and **e**) and ring enhancement (arrow in **f**). This is a pyogenic abscess

There are a number of infectious agents which have a predilection for the thalamus such as viral (influenza A, parainfluenza) and bacterial (*Mycoplasma pneumoniae*, *Mycobacterium tuberculosis*) frequently with the involvement of the brainstem, basal ganglia, cerebellum, and periventricular white matter. As in other locations, a thalamic abscess can complicate bacterial infection and present the same semiology as in other brain regions with diffusion restriction and ring enhancement (Fig. 14). A possible pitfall in this case would be a subacute haematoma, which is particularly common in this location in patients with high blood pressure.

Another entity that affects the thalamus, particularly the pulvinar, is the Creutzfeld-Jacob disease (CJD) or spongiform encephalopathy, a disease caused by a prion and resulting in rapidly progressive dementia. In CJD, MRI depicts T2WI/FLAIR hyperintensity as well as diffusion restriction of grey matter including the basal ganglia, thalami, and cortex (mostly frontal and temporal lobes). Brain atrophy rapidly ensues. The “pulvinar sign” or “hockey stick sign” (hyperintensity involving the pulvinar and dorsomedial thalamic nuclei bilaterally on FLAIR) may be seen. There is typically no enhancement, and white matter is usually not involved. The differential diagnosis includes hypoxic-ischaemic encephalopathy, arterial ischaemia, Wilson’s disease, and HIV1 encephalopathy [16].

### Epilepsy

**Fig. 15:**
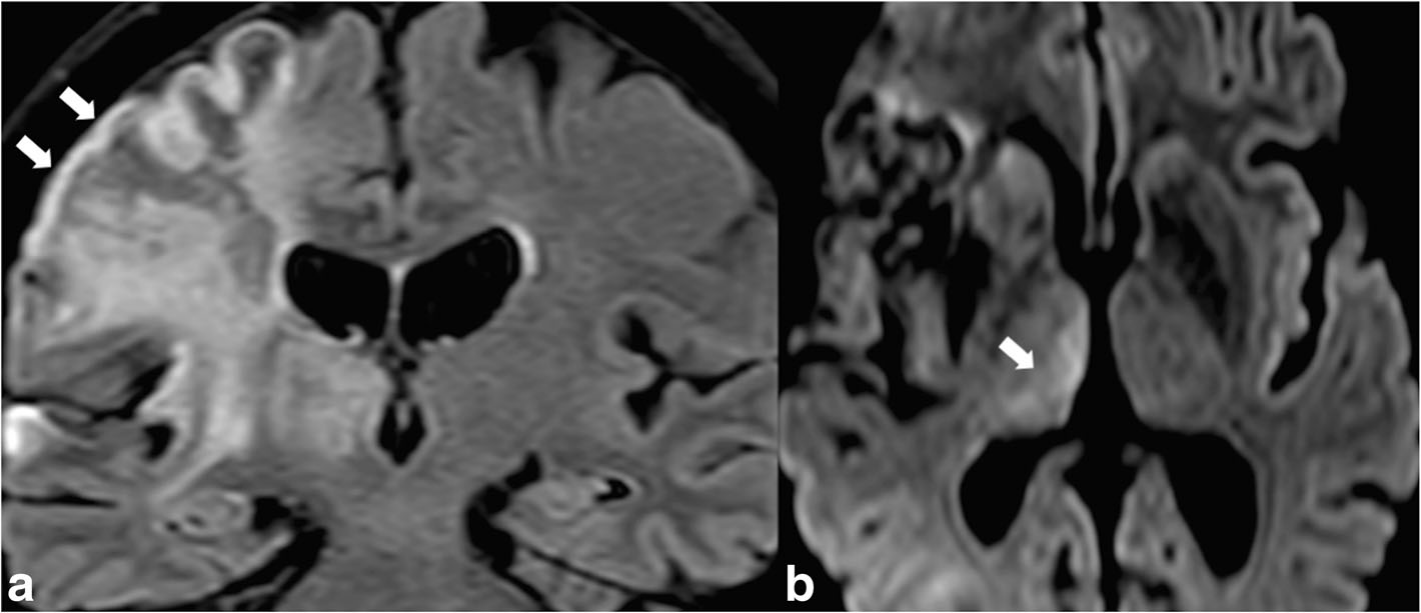
FLAIR hyperintensity in the right temporal and insular regions (arrows in **a**) reflecting brain ischaemia. An epileptic seizure ensued due to cortical involvement, which in turn resulted in high signal intensity on DWI b1000 in the right thalamus (arrow in **b**) as a consequence

Following status epilepticus, but also in the case of an isolated episode or cluster of epilepsy, MRI may show abnormalities in the thalamus as T2WI hyperintensity and diffusion restriction in the early postictal phase [17]. These abnormalities are usually reversible (Fig. 15).

### Electrode mapping

**Fig. 16:**
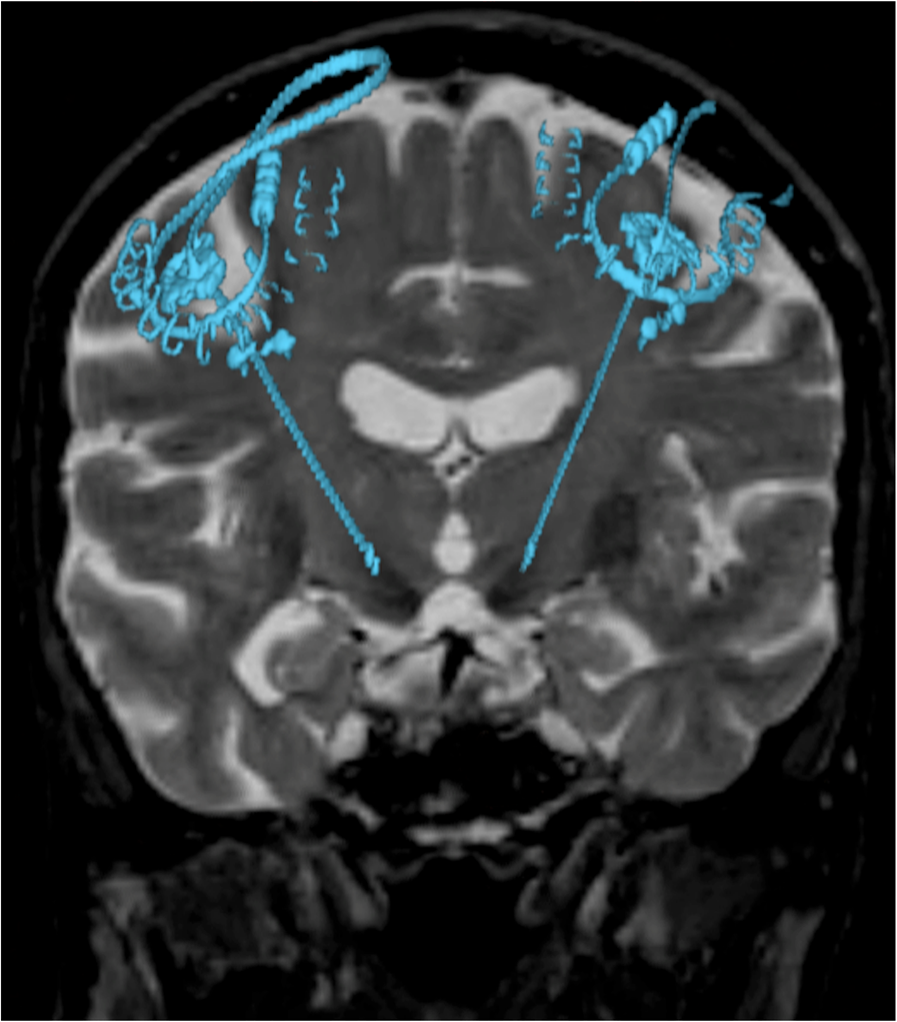
Coronal 3D T2WI fused with CT image shows electrodes of deep brain stimulation (DBS) in both subthalamic nuclei

MRI is used in the 3D mapping of the brain for stereotactic neurosurgery due to its remarkable soft tissue resolution. This is usually performed to guide placement of deep brain stimulation (DBS) electrodes in parkinsonism [18, 19] for stimulation of the thalamic *nucleus ventralis intermediu*s (Fig. 16) and also in the treatment of epilepsy.
